# Expression of the RON receptor tyrosine kinase and its association with gastric carcinoma versus normal gastric tissues

**DOI:** 10.1186/1471-2407-8-353

**Published:** 2008-11-28

**Authors:** Donghui Zhou, Gang Pan, Chen Zheng, Jingjing Zheng, Liping Yian, Xiaodong Teng

**Affiliations:** 1 Department of oncology, The First Affiliated Hospital of College of Medicine of Zhejiang University, Hangzhou, 310003, PR China; 2 Department of Surgery, Lishui Central Hospital, Lishui, 323000, PR China

## Abstract

**Background:**

Recepteur d'origine nantais (RON) is a receptor tyrosine kinase that is activated by a serum-derived, macrophage stimulating protein (MSP) growth factor and is expressed in many malignant tumors. The aim of the present study was to reveal the protein expression profile of RON and its relationship with clinicopathological characteristics of gastric carcinoma and prognosis.

**Methods:**

Gastric carcinoma tissue from 98 patients, along with 29 specimens of paraneoplastic tissue and 10 specimens of normal gastric mucosa, were examined by immunohistochemistry (IHC). Western blot analysis of 19 samples of gastric carcinoma tissue and corresponding paraneoplastic tissue, 8 specimens of normal gastric mucosa, and 2 specimens of normal lymph node samples also detected expression of a splice variant of RON, RONΔ165. All samples obtained were accompanied by patient follow-up data that ranged from 3 to 89 months (median time: 22 months).

**Results:**

The rate of positive RON expression differed significantly between gastric carcinoma tissues [56.1%, (55/98)] and paraneoplastic tissues [25.6%, (8/29)] (p = 0.007). In contrast, RON expression was absent in normal gastric mucosa samples. RON expression positively correlated with the invasive depth of the tumor (p = 0.019), perigastric lymph nodes metastasis (p = 0.019), and TNM stage (p = 0.001). However, RON expression was independent of tumor growth pattern according to Bormann criteria (p = 0.209), histopathological grade (p = 0.196), and incidence of distant metastasis (p = 0.400). RON expression was not related to a patient's survival rate (p = 0.195). RONΔ165 was strongly expressed in fresh gastric carcinoma tissue, corresponding paraneoplastic tissue, and perigastric lymph nodes with metastatic carcinoma. In contrast, expression of RONΔ165 was not observed in normal gastric mucosa and normal lymph node tissue samples.

**Conclusion:**

RON expression is significant in gastric carcinoma tissue and corresponding paraneoplastic tissue, but is not expressed in normal gastric mucosa. Expression of RONΔ165 was similarly observed in gastric carcinoma tissue and in metastases present in lymph node tissues. We hypothesize that RON and its splice variant play an important role in the occurrence, progression, and metastasis of gastric carcinoma, and therefore may represent a useful marker to evaluate the biological activity of gastric carcinoma.

## Background

Morbidity from gastric carcinoma is increasing each year, while the age of onset is decreasing. In China, gastric carcinoma is the top malignant tumor in both categories of morbidity and mortality [1]. Recepteur d'origine nantais (RON) is a receptor tyrosine kinase (RTK) that belongs to the MET proto-oncogene family. Recent studies of a RON knockout mouse model shows that complete disruption of the RON gene is embryonic lethal [2], demonstrating that RON is essential in embryonic development. RON is activated by a serum-derived, macrophage stimulating protein (MSP) growth factor, and studies have shown that RON is expressed in many malignant tumors and plays a role in their occurrence and progression.

Zhou et al. [3] found that RON is strongly expressed in colorectal carcinomas, and they identified three splicing variants of RON (RONΔ160, RONΔ165, and RONΔ155). The expression of RON and its variants was associated with the progression of colorectal carcinoma. Other studies have shown that the expression of RON and its splice variants can precipitate colon epithelial cell colony formation and increase their viability. When expression of RON and its splice variants were targeted by siRNA in rectal tumor cell strains, proliferation and metatasis were significantly inhibited, concomitant with increased apoptosis. Based on these data, RON and its splice variants are hypothesized to have an important role in the occurrence, progression, and metastasis of rectal cancer [4]. Similarly, studies of RON expression in both breast and bladder cancer tissues has shown increased levels of RON expression and a correlation with histological grade [5,6].

To our knowledge, the role of RON in gastric cancer has only been studied by Collesi et al [7]. In this study the splice variant of RON, RONΔ165, was detected in the gastric carcinoma cell strain, KATO-III. RONΔ165 was shown to enhance the invasion of gastric cancer cells, indicating a role for the splice variant of RON in the malignant transformation of gastric cells to a carcinoma. In this study, we examine the expression of RON in the gastric carcinoma tissue of 98 patients using the Envision immunohistochemistry (IHC) method. To examine expression of RONΔ165, Western blotting of fresh gastric carcinoma tissue was performed. The patients enrolled in our study were monitored between 3 and 89 months post-operation to investigate the association of RON expression, including its splice variant, to the clinicopathological characteristics of gastric carcinoma and prognosis. Due to the complex pathogenesis of gastric carcinoma, therapeutic efficacy has not proven to be very effective. Thus, it is important to investigate the pathogenesis of gastric carcinoma to find new therapeutic targets.

## Methods

### Patient samples

Paraffin embedded tissue samples from 98 patients who underwent surgery for pathologically confirmed gastric carcinoma in the First Affiliated Hospital of College of Medicine of Zhejiang University between 1998 and 2003 were obtained. Gastric samples were used with the consent of the patients and permission of the Ethics Committees of Hospitals. Seventy men and 28 women, ranging in age from 21 to 76 years (median age 58 years), were included in this study. Patient diagnosis included 78 cases of sinus ventriculi carcinoma, 16 cases of gastric cardia carcinoma, and 4 cases of total gastric carcinoma. Post-surgery pathological reports diagnosed 2 well-differentiated adenocarcinomas, 21 moderately differentiated adenocarcinomas, 69 poorly differentiated adenocarcinomas, 1 signet-ring cell carcinoma, 2 mucinous adenocarcinomas, and 3 severe dysplasia with cancerization. According to UICCTNM (1998), the histological grade of the samples was determined to include 26 stage I cases, 14 stage II cases, 30 stage III cases, and 28 stage IV cases. The follow-up rate for our patients was 92%, with 8 patients not available to complete the follow-up. The follow-up period was between 3 and 89 months (median time of 22 months) following surgery. Ten samples of normal gastric mucosa were examined as controls. Nineteen samples of fresh gastric carcinoma tissue, paraneoplastic tissue, and positive perigastric lymph node tissue were collected for Western blot analysis. Ten samples of normal gastric mucosa were also included as controls.

### Immunohistochemistry

Paraffin samples were sectioned into 4 μm thick slices which were deparaffinized, hydrated and incubated for 20 min in 0.3% H_2_O_2 _to inhibit endogenous peroxidase activity. For antigen retrieval, samples were incubated with 0.1 M citrate buffer (pH 6.0) and heated in a pressure cooker for 8 min. After rinsing in TBS, the slides were incubated with 3% normal donkey serum (NDS) in TBS for 20 min to prevent non-specific binding of the first antibody. Anti-RON rabbit monoclonal antibody Ron β (C-20) (Santa Cruz Biotechnologies, USA) was used at 1:400. The Envision kit (DAKO) was used according to the manufacturer's directions, and bound antibody was detected using DAB. Samples were also co-stained with hematoxylin. Samples were observed under a light microscope and a PBS-only staining sample was used as a background control.

### Evaluation criteria for RON detection by immunohistochemistry

The assessment of the grade of staining was determined using a blinded evaluation procedure by experienced pathologists. High-power fields (400×) using standard light microscopy were divided into ten sections which were randomly scored. Tumor cells with buffy particles in the intracytoplasm were scored as positive, and the percentage of positive tumor cells and staining intensity for each sample was recorded. Tumor cells that did not stain for RON expression were scored as negative, cells that weakly stained were scored as 1, moderate staining was scored with a 2, and strongly staining samples were scored as 3. Of the positive tumor cells detected,< 5% were negative, between 5 and 24% were scored as 1, 25–49% were scored with a 2, and > 50% were scored with a 3. The cumulative score of the tumor cells identified in a sample determined a sample's final score. Based on the final score, the tumor tissue was determined to be negative (score of 0–2), or positive (score of 3–6) for RON expression.

### Protein immunoblotting

Fifty micrograms of total protein for each sample was separated by electrophoresis on a 10% polyacrylamide gel and transferred to PVDF membrane (Millipore). Detection of RON protein was performed using an anti-RON rabbit monoclonal antibody Ron β (C-20) (Santa Cruz Biotechnologies, USA) (1:400). Antibody binding was visualized using an anti-rabbit antibody conjugated to horseradish peroxidase and an Enhanced chemiluminescence kit (ECL) (Biological Industries, Inc. Israel). β-actin was used as a loading control.

### Statistical analysis

SPSS 13.0 statistical software was used to identify the statistical significance of our data using fourfold tables or the R × C table χ^2 ^test. Survival curves were evaluated using the Kaplan-Meier method and comparisons of survival rates were tested using a Log-rank test.

## Results

### RON expression in gastric tissue and paraneoplastic tissue

**Figure 1:**
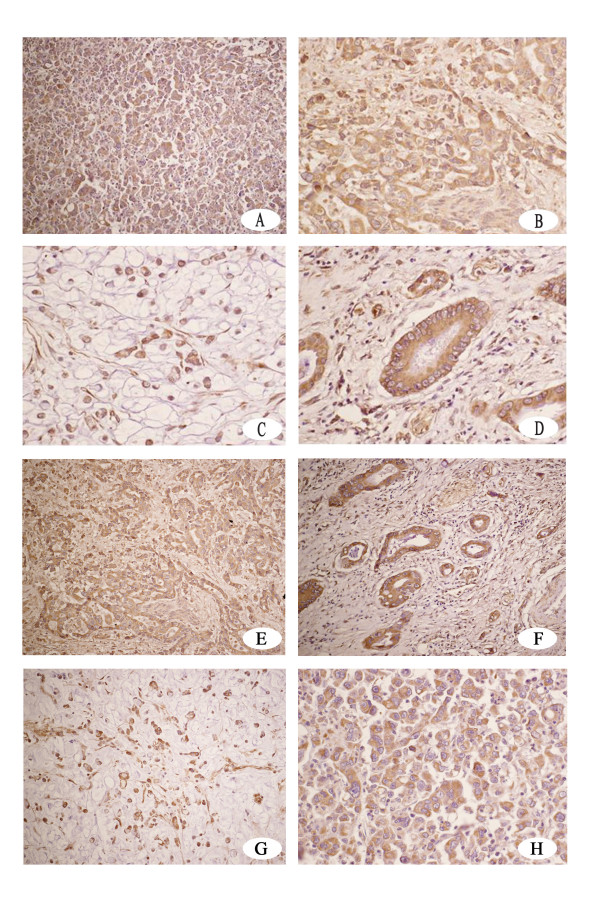
**Detection of RON expression by immunhistochemistry**. A, H) Detection of RON in a poorly differentiated gastric adenocarcinoma sample. 200× and 400 × magnification. B, E) Magnified view of RON detection in a sample of poorly differentiated gastric adenocarcinoma. 400× and 200× magnification. C) Detection of RON in a sample of signet-ring cell carcinoma. 400× magnification. D) Detection of RON in a sample of moderately differentiated gastric adenocarcinoma. 400× magnification. F) Detection of RON in a sample of moderately differentiated gastric adenocarcinoma. 200× magnification. G) Detection of RON in a sample of signet-ring cell carcinoma. 400× magnification.

RON protein was observed in the cytolymph, but not in the nucleolus, of gastric carcinoma cells and typically appeared as a buffy granulo-staining (Fig. 1). The percentage of positive tumor cells and the staining intensity for each sample was recorded. For the 98 patient samples evaluated, RON expression was observed in 56.1% of the gastric carcinoma tissues (55/98), but not in samples of normal gastric mucosa. In gastric carcinoma paraneoplastic tissue, which was collected from the gastric mucous layer 0.5 cm away from the gastric carcinoma specimens, RON protein was observed in 25.6% of samples (8/29). These data represent a significant statistical difference in RON protein expression between gastric carcinoma tissue and its associated paraneoplastic tissue (χ^2 ^= 7.290, p = 0.007).

### RON protein expression in relation to the pathology of gastric carcinoma

RON protein expression was not found to be associated with gender, age, or diseased region (*p *> 0.05). However, in gastric carcinoma tissues, the deeper a carcinoma cell infiltrated, the more strongly RON protein was expressed (*p *= 0.019). RON protein expression was also found to positively correlate with perigastric lymph node metastasis and clinical pathology stage with statistical significance (*p *= 0.019, *p *= 0.001) (Table 1). In contrast, there were no significant statistical differences in RON expression with the pathology grading, Borrmann type, or presence of metastasis. However, it was observed that RON expression was stronger in the Borrmann III/IV group (63.6%) than in the Borrmann I/II group (53.8%), stronger in the distant metastasis group (68.2%) than in the non-distant metastasis group (52.6%), and stronger in the histologically lower differentiated group (52.4%) than in the moderately and highly differentiated histology group (39.1%). There were no significant statistical differences for these comparisons (p = 0.209; p = 0.400; p = 0.196), respectively (Table 1).

**Table 1:** Relationship of RON expression to pathology parameters

	RON	RON	Χ^2^	p
	(+) (%)	(-)		
**Invasive Depth**				
Early gastric cancer	5 (29.4)	12		
Infiltrated to muscular layer	15 (51.7)	14	7.795	p = 0.019
Infiltrated to serous membrane	35 (67.3)	17		
				
**TNM Stage**				
Stage	14 (35.0)	26	12.245	p = 0.001
Stage	41 (70.7)	17		
				
**Metastases to Lymph Nodes**				
N_0_	11 (37.9)	18		
N_1–3_	44 (63.8)	25	5.535	p = 0.019
				
**Borrmann Grade**				
I/II group	14 (53.8)	12		
III/IV group	35 (63.6)	20	0.708	p = 0.400
Others	6	11		
				
**Histology Grade**				
Moderately & highly differentiated	9 (39.1)	14	1.577	p = 0.209
Less differentiated *	39 (54.2)	33		
Others**	0	3		
				
**Distant Metastasis**				
M_0_	40 (52.6)	36	1.675	p = 0.196
M_1_	15 (68.2)	7		

*Includes poorly differentiated adenocarcinoma, signet-ring cell carcinoma, mucinous adenocarcinoma; ** Severe dysplasia with cancerization.

### RON expression and survival rates

**Figure 2:**
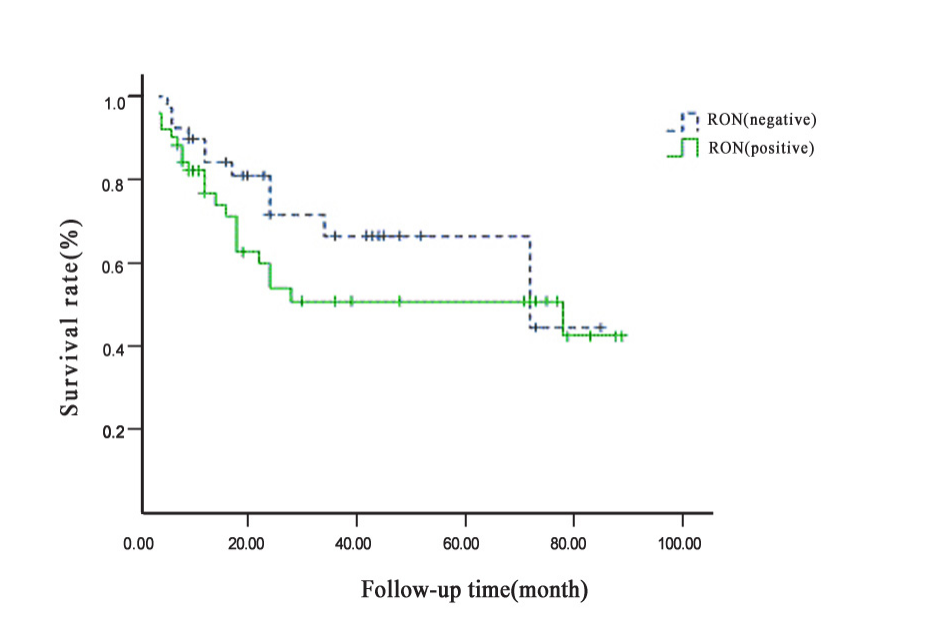
Survival curve for RON+ versus RON-patients.

For the 90 patients enrolled in this study that were followed post-operationally, no significant statistical difference in survival rates was found between patients with RON-positive gastric carcinoma samples versus RON-negative gastric carcinoma samples (Fig. 2).

### Detection of the RONΔ165 splice variant

**Figure 3:**
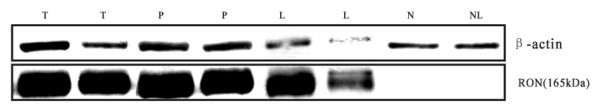
**Western blot analysis of RON expression in gastric carcinoma tissue, paraneoplastic tissue, and perigastric lymph nodes**. Duplicate samples of gastric carcinoma tissue (T), paraneoplastic tissue (P), and metastatic lymph node (L) tissues were analyzed for expression of RONΔ 165 (165 kDa). Normal gastric mucosa (N) and normal lymph node (NL) tissue samples were compared. β-actin (44 kDa) was used as a loading control.

**Figure 4:**
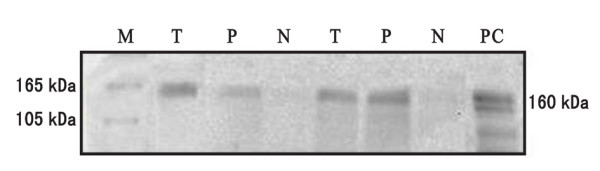
**Western blot analysis of RON expression in gastric carcinoma tissue, paraneoplastic tissue, and normal gastric mucosa**. Duplicate samples of gastric carcinoma tissue (T) and paraneoplastic tissue (P) were analyzed for expression of RONΔ165 (165 kDa) and compared with normal gastric mucosa (N) tissue samples. Marker (M) and positive control (PC) (160 kDa) were included.

In the 19 samples of fresh gastric carcinoma tissues collected, along with corresponding paraneoplastic tissues, and pathologically confirmed perigastric lymph nodes with metastatic carcinoma, the splice variant of RON, RONΔ165, was expressed. In contrast, no expression of RON protein was observed in normal gastric mucosa or normal lymph node tissue (Fig. 3, 4).

## Discussion

In the current study, we show by both IHC and Western blot, that RON expression is not detectable in normal gastric mucosa. These observations are consistent with results from Okino et al [8]. However, staining of RON in carcinoma tissues and corresponding paraneoplastic tissues by IHC showed significant expression of RON relative to normal gastric mucosa samples. Western blot analysis provided more specific information regarding RON expression with detection of RONΔ165 in gastric carcinoma tissues, corresponding paraneoplastic tissues, and lymph nodes with carcinoma metastases. These data indicate that expression of full-length RON, as well as a variant of RON, are expressed during the process of malignant transformation of gastric epithelial cells. Based on these data and work in other studies on the role of RON in tumorigenic phenotypes, we hypothesize that RON may act as an oncogene to promote the occurrence, progression, invasion, and metastasis of gastric cancer.

Previous studies have shown that expression of RON correlates with the invasion of tumor cells. Maggiora et al [9] reported that RON was expressed in 55% of ovarian cancer tissues. Furthermore, if an ovarian cancer cell line that strongly expresses RON is induced by MSP, tumor growth and invasion is significantly enhanced. Based on these data, it was hypothesized that RON plays a role in the progression of ovarian cancer. Similarly, Chan et al [10] showed that expression of RON promoted the growth and malignant transformation of skin papillomas, and Cheng et al [6] showed that strong expression of RON in bladder cancer cell lines correlated with increased proliferation and apoptosis inhibition. The latter study also demonstrated that the expression of RON in gastric carcinoma positively correlated with the invasive depth of the gastric cancer cell and lymph nodes metastasis, and the level of expression increased significantly with higher clinical pathologies of gastric cancer. Therefore, strong expression of RON in gastric cancer tissues may serve as a marker to indicate a conversion of normal cells to a malignant phenotype, which associated with increased tumor cell invasion and metastasis, would be associated with a poor prognosis for long-term survival. The proximity of gastric cancer tissue to the epithelial dysplasia of the stomach results in the susceptibility of the epithelial dysplasia of the stomach to precancerous lesions. Therefore, by reporting the expression of RON in paraneoplastic tissue, which was collected ~0.5 cm away from the gastric carcinoma samples, we are providing data to support our hypothesis that RON plays an important role in promoting gastric cancer and may serve as a marker for the conversion of tissues to a malignant phenotype.

In previous studies looking at the relationship between RON expression and survival rates, Lee et al [11] found that MET and RON were independent prognostic factors of long-term recurrence of breast cancer, and that specimens positive for MET and RON expression had a significantly lower 10-year disease-free survival rate. Similarly, Cheng et al [6] reported a lower survival rate for bladder cancers positive for both MET and RON. In contrast, we found that the survival rate of patients positive for RON expression was not lower than the survival rate for the RON-negative patient group. It is possible that the survival rate of patients with gastric cancer can be affected by many factors, and our follow-up data was not sufficient to provide additional insight into which factors might be important.

## Conclusion

The results of this study show that RON is expressed in gastric cancer, but not in normal gastric mucosa. Furthermore, the expression rate of RON in gastric cancer positively correlates with the invasive depth, the clinical pathologic stage, and extent of lymph node metastasis. We hypothesize that RON plays an important role in the occurrence, progression, invasion, and metastasis of gastric cancer, and therefore could be an important marker in assessing the biological behavior of gastric cancer. The results of our studies provide a foundation from which to further identify the mechanistic details of the role of RON in gastric cancer in order to identify RON as a therapeutic target in gastric cancer.

## Competing interests

The authors declare that they have no competing interests.

## Authors' contributions

ZDH carried out study design, final data analysis, drafted the manuscript and revisions to the manuscript. PG, ZC and ZJJ participated in the study concept and the primary data analysis. YLP and TXD performed the IHC analysis. PG was responsible for the Western blot analysis. All authors read and approved the final manuscript.

## Pre-publication history

The pre-publication history for this paper can be accessed here:


